# Towards *in vivo* photoacoustic human imaging: Shining a new light on clinical diagnostics

**DOI:** 10.1016/j.fmre.2023.01.008

**Published:** 2023-02-14

**Authors:** Zhiyang Wang, Fei Yang, Wuyu Zhang, Kedi Xiong, Sihua Yang

**Affiliations:** a MOE Key Laboratory of Laser Life Science & Institute of Laser Life Science, College of Biophotonics, School of Optoelectronic Science and Engineering, South China Normal University, Guangzhou 510631, China; b Guangdong Provincial Key Laboratory of Laser Life Science, College of Biophotonics, School of Optoelectronic Science and Engineering, South China Normal University, Guangzhou 510631, China

**Keywords:** Photoacoustic imaging, Clinical diagnostics, Biomedical optics, Human imaging, Multiscale imaging

## Abstract

Multiscale visualization of human anatomical structures is revolutionizing clinical diagnosis and treatment. As one of the most promising clinical diagnostic techniques, photoacoustic imaging (PAI), or optoacoustic imaging, bridges the spatial-resolution gap between pure optical and ultrasonic imaging techniques, by the modes of optical illumination and acoustic detection. PAI can non-invasively capture multiple optical contrasts from the endogenous agents such as oxygenated/deoxygenated hemoglobin, lipid and melanin or a variety of exogenous specific biomarkers to reveal anatomy, function, and molecular for biological tissues *in vivo*, showing significant potential in clinical diagnostics. In 2001, the worldwide first clinical prototype of the photoacoustic system was used to screen breast cancer *in vivo*, which opened the prelude to photoacoustic clinical diagnostics. Over the past two decades, PAI has achieved monumental discoveries and applications in human imaging. Progress towards preclinical/clinical applications includes breast, skin, lymphatics, bowel, thyroid, ovarian, prostate, and brain imaging, etc., and there is no doubt that PAI is opening new avenues to realize early diagnosis and precise treatment of human diseases. In this review, the breakthrough researches and key applications of photoacoustic human imaging *in vivo* are emphatically summarized, which demonstrates the technical superiorities and emerging applications of photoacoustic human imaging in clinical diagnostics, providing clinical translational orientations for the photoacoustic community and clinicians. The perspectives on potential improvements of photoacoustic human imaging are finally highlighted.

## 1 Introduction

### 1.1 Photoacoustic imaging

**Fig. 1:**
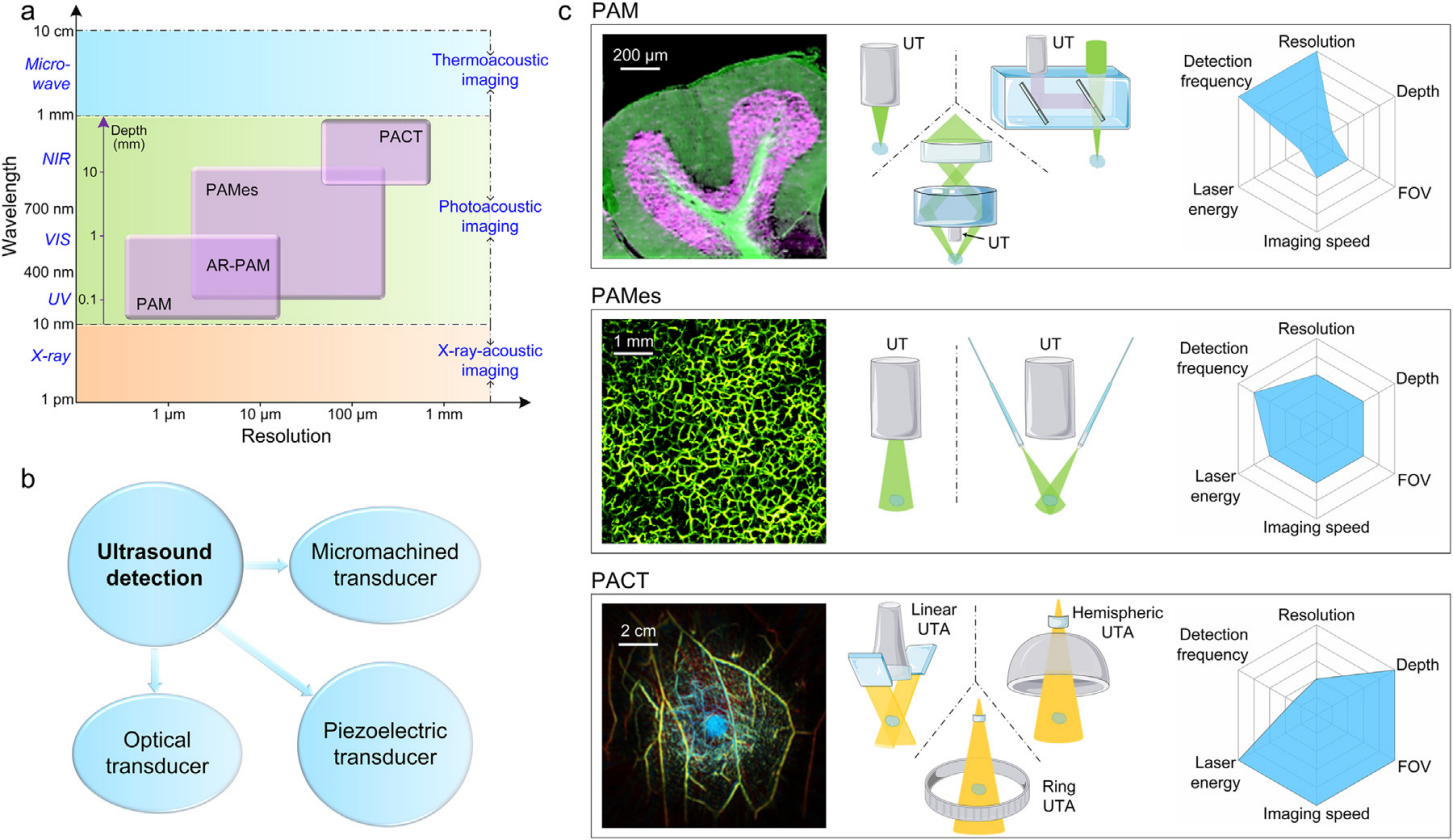
**Different configurations and parameters of photoacoustics.** (a) Photoacoustic effect under different pulsed illumination sources, and the spatial resolution in photoacoustic imaging. According to the wavelength of the illumination sources, it can be specifically divided into thermoacoustic imaging, photoacoustic imaging, and X-ray-acoustic imaging. (b) Different ultrasound detection techniques for photoacoustic imaging. (c) Different configurations and imaging parameters for different optical excitations and acoustic receptions [72,109,191].

Photoacoustic (PA) imaging (PAI) is a hybrid imaging technique with clinical translational value [1], [2], [3], [4], [5], [6], [7], [8], [9], [10], [11], which transmits information through ultrasound to characterize three-dimensional distribution of optical absorption of biological tissues. By combining the high-contrast optical imaging and the deep-penetration ultrasonic imaging, PAI has significant potential applications in biomedicine [12], [13], [14], [15], [16]. On the one hand, compared with pure optical imaging techniques (*e.g.* OCT, two-photon imaging, photothermal microscopy) [17], [18], [19], [20], [21], PAI uses the received ultrasonic signal to reconstruct the PA image, and the scattering of ultrasonic signal in biological tissues is 2∼3 orders of magnitude lower than that of optical signal [1,22]. As a result, PAI enables deep imaging of complex multilayer human tissues *in vivo*. On the other hand, compared with pure ultrasonic imaging technique, PAI can flexibly select visible light, near-infrared light, or even electromagnetic waves in the microwave range for illumination according to the optical characteristics of the targeted biological tissues, and finally reconstruct the light-absorption distribution in biological tissues with excellent imaging contrast and resolution. As shown in Fig. 1a, depending on the types of pulsed illumination sources, the applications of photoacoustic effect include X-ray-induced acoustic computed tomography [23,24], microwave-induced thermoacoustic imaging [25,26], and PAI [1], in which PAI is the most mature and widely used branch. For the working mode of seeing biological tissues by listening to the sound of light, ultrasonic detection is an essential part of the PAI system [27]. The advancement of ultrasound detection technology has played a significant role in promoting the translational applications of PAI. Current ultrasonic detection methods include traditional piezoelectric transducer, micromachined transducer, and all-optical detection technology (Fig. 1b) [28]. Depending on the practical experimental backgrounds and the geometric configurations of the system, suitable ultrasonic detection methods can be adopted by researchers. At present, piezoelectric transducers with mature technology are mostly used in clinical photoacoustic human imaging. In addition, as a hybrid imaging technique, PAI is naturally married with conventional clinical imaging techniques to integrate optical, ultrasonic, and magnetic multimodal imaging technology, providing comprehensive morphological structures and multifunctional physiological conditions, which makes it of great practical value in the diagnosis of human diseases.

With the advent of PAI technology, review articles on PAI have also been published, summarizing physical theories, basic research, system improvement, and biomedical applications [29], [30], [31], [32], [33], [34], [35]. Further, benefiting from technological and engineering advances, PAI has demonstrated tremendous clinical translational value and feasibility. Given that most of the previous reviews were mixed presentations of basic theories, animal experiments, human imaging studies, or specific application categories [34,[36], [37], [38], [39], [40]]. To present the photoacoustic community and clinicians with a more comprehensive understanding of the application progress of photoacoustic human imaging *in vivo*, the breakthrough research in the engineering and preclinical/clinical applications of photoacoustics is summarized in this review. All the cases are from *in vivo* human imaging studies, skipping basic studies in animal models, to draw a preliminary manual for the application of photoacoustic human imaging. For PAI systems, different configurations of optical excitation and acoustic detection determine the parameters of imaging resolution and penetration depth (Fig. 1c). Although some international research groups have different classifications of PAI, in general, based on spatial resolution, PAI systems are classified as photoacoustic microscopy (PAM) [41], photoacoustic mesoscopy (PAMes) [42], and photoacoustic computed tomography (PACT) [43,44]. In the PAM system, short-wavelength lasers (ultraviolet or visible light) with tens-of-nj energy are usually used for optical illumination, and a high-frequency ultrasonic transducer is used to receive the PA signal. Both laser illumination and ultrasound detection are focused, and the optical/acoustic dual foci are usually configured as coaxial and confocal to enhance the imaging sensitivity. The PAM system can be further defined as optical-resolution PAM (OR-PAM) and acoustic-resolution PAM (AR-PAM) according to the actual sizes of the optical/ultrasound foci. Although PAM has limited penetration depth and field of view, its resolution is the best in PAI, providing lateral resolution ranging from a few hundred nanometers to tens of micrometers. PAM can be used for high-resolution non-invasive imaging of organelles and capillaries in preclinical and clinical applications [4,45]. For example, ultraviolet photoacoustic microscopy (UV-PAM) holds great potential as an intraoperative histopathological method for tumor margin identification [46,47], and real-time PAM can depict the microcirculation of human subcutaneous capillaries [48]. As another configuration, PACT usually uses optical fiber bundles to flexibly set illumination for different imaging sites, and uses ultrasonic transducer arrays to receive ultrasonic signals in parallel [13]. Near-infrared laser is commonly used in PACT to achieve deep tissue penetration. PACT has real-time imaging speed and a larger field of view, so PACT is mainly used for macroscopic imaging of deep subcutaneous arteries and superficial organs [43]. It is worth noting that PACT is very easy to be combined with clinical ultrasound probes to realize PA and ultrasound dual-mode imaging [49,50], which can provide complementary information such as tissue absorption and blood oxygen for clinical ultrasonography. As a bridge between the spatial resolution of PAM and PACT, PAMes can acquire a few of millimeters with deep tissue penetration and a few tens of micrometers with resolutions (Fig. 1a) [42]. PAMes has successfully developed emerging applications in biomedicine. Now, PAMes has depicted the fine anatomical morphology of human skin from epidermis to subcutaneous tissue, which provides researchers and clinicians a new way of non-invasively seeing detailed skin structures [51].

### 1.2 Technology advances in photoacoustic human imaging

#### 1.2.1 Quantitative chromophore analysis

Multiple endogenous chromophores in tissues can be used as markers for disease diagnosis and treatment. For example, melanin, lipid, and collagen are closely related to melanoma, atherosclerosis, and muscular diseases, respectively. Multispectral photoacoustic imaging can realize quantitative visualization of different chromophores based on spectral analysis technology. Spectral analysis technology can extract absolute chromophore concentration from multi-wavelength photoacoustic images. It is realized by spectral unmixing based on the absorption coefficients for each chromophore at different wavelengths. The spectral unmixing was performed on PA images using the following listed procedure [52]:

(1) $$\left[C_{1}C_{2}\cdots C_{n}\right]=\left[PA_{1}PA_{2}\cdots PA_{i}\right]\cdot M^{T}\cdot {\left[M\cdot M^{T}\right]}^{-1}\;K$$

(2) $$M=\left[\begin{array}{cccc} \mu_{\lambda_{1}}^{1} & \mu_{\lambda_{2}}^{1} & & \mu_{\lambda_{i}}^{1}\\ \mu_{\lambda_{1}}^{2} & \mu_{\lambda_{2}}^{2} & \cdots & \mu_{\lambda_{i}}^{2}\\ \vdots & & \ddots & \vdots \\ \mu_{\lambda_{1}}^{n} & \mu_{\lambda_{2}}^{n} & \cdots & \mu_{\lambda_{i}}^{n} \end{array}\right]$$

Where *K* is the proportionality coefficient, $C_{n}$ is the concentration of the n^th^ component, $PA_{i}$ is the normalized and median filtered photoacoustic signal at the i^th^ wavelength, and $\mu_{\lambda_{i}}^{n}$ is the normalized light absorption coefficient of the n^th^ component at the i^th^ wavelength. Theoretically, this method requires at least more than two wavelengths of photoacoustic signals for accurate spectral analysis, and using more excitation wavelengths can reduce the influence of measurement error.

#### 1.2.2 Photoacoustic functional parameters

PAI can not only provide high-resolution structural images but also reveal functional information of biological tissues. Quantitative functional parameters are expected to evolve into new diagnostic criteria for diseases. For example, photoacoustic viscoelasticity obtains the mechanical properties of tissue through the phase information of the acoustic signal [53,54]. This method can simultaneously quantify the elasticity and viscosity of biological tissue by measuring the rise time of the photoacoustic signal corresponding to the displacement and the phase difference relative to the excitation. When the pulsed laser irradiates the tissue, the rise time $t_{max}$ required for the displacement to reach maximum amplitude is related to elasticity of medium. The displacement reaches its peak at $t_{max}=R/\sqrt{E/\rho }$. When the mass density of the biological tissue ρ and the waist radius of the Gaussian laser beam R are known, the elastic modulus E can be estimated by [55]:

(3) $$E=\rho {\left(R/t_{max}\right)}^{2}$$

On the other hand, after being irradiated by the pulsed laser, the viscoelastic properties of tissue make it not instantaneously respond, but gradually deform. Therefore, in the case of laser-induced photoacoustic wave, the response of photoacoustic wave to the excitation laser stimulus has a phase delay. The phase delay δ of the PA wave to the stress is given by tanδ=ηω/E, where ω is the modulation frequency. Photoacoustic viscoelasticity aids in the search for novel markers of tumor foreign bodies and vulnerable plaques.

The photoacoustic oxygen saturation measurement is based on the absorption difference between oxygenated hemoglobin and deoxygenated hemoglobin. The total blood absorption coefficient $\mu_{a}\left(\lambda_{i}\right)$ ($cm^{-1}$) can be expressed as [56]:

(4) $$\mu_{a}\left(\lambda_{i}\right)=\varepsilon_{\text{HbR}}\left(\lambda_{i}\right)\left[\text{HbR}\right]+\varepsilon_{\text{Hb}O_{2}}\left(\lambda_{i}\right)\left[\text{Hb}O_{2}\right]$$

Where [HbR] and [HbO_2_] are the concentrations of the two forms of hemoglobin, and $\varepsilon_{HbR}\left(\lambda_{i}\right)$ and $\varepsilon_{HbO_{2}}\left(\lambda_{i}\right)$ are the molar extinction coefficients of HbR and HbO_2_ at wavelength $\lambda_{i}$. After quantitative spectral analysis, the photoacoustic oxygen saturation $SO_{2}$ is calculated as:

(5) $$SO_{2}=\frac{\left[\text{Hb}O_{2}\right]}{\left[\text{Hb}O_{2}\right]+\left[\text{HbR}\right]}$$

When the tissue has early lesions, although there is no obvious abnormality in the anatomical structures, the local oxygen demand of the lesions increases, so the oxygen saturation is expected to be a key parameter for early diagnosis of lesions. At present, with the compensation and optimization of optical fluence in biological tissues, the quantitative analysis of photoacoustic oxygen saturation in deep tissues is more accurate, which is of great significance in clinical diagnosis and screening.

#### 1.2.3 Fluence compensation

In 3D photoacoustic imaging, wavelength-dependent optical attenuation, non-uniform incident optical fluence, and the difference in sound speed-of-sound velocity all limit imaging depth and can weaken the accuracy of structural and functional imaging [57,58]. For example, the non-uniform optical fluence affects oxygen saturation and composition analysis, and the variation in the speed-of-sound between different tissues can also lead to distortions of the position and shape of the reconstructed images. Compensating for the heterogeneity of speed-of-sound and depth-dependent optical fluence can effectively modify the structural and functional parameters of photoacoustic imaging, thereby enhancing the reliability of clinical human imaging. According to the optical excitation structure, Optical transmission properties, and the optical properties of the target imaging tissue, the nonuniform incident optical fluence in the tissue can be reasonably assumed. The most used methods are to establish 3D modeling of the photon transportation through Beer-Lambert law, Monte Carlo modeling, etc., to enhance the accuracy of functional imaging and the signal-to-noise ratio in low-fluence regions. But this method requires a lot of computation and requires some delay even when using GPU for acceleration. To address this problem, real-time interleaved spectroscopic compensation for wavelength-dependent laser fluence is proposed [59], which requires no additional equipment and delays, and is expected to be transferred to human imaging *in vivo*.

#### 1.2.4 Motion correction

The transfer of photoacoustic imaging from basic research to clinical (preclinical) research will introduce more motion artifacts, such as in photoacoustic microscopy and photoacoustic mesoscopic imaging, due to the slow scanning speed, the imaging results will be affected by human movement, breathing, or the influence of heartbeat. In multispectral photoacoustic tomography, when switching imaging of different excitation wavelengths at the same position, it is also affected by motion artifacts. In general, motion artifacts will reduce the effective resolution and quantitative analysis of photoacoustic results. To provide reliable and high-quality imaging results in photoacoustic human imaging, motion-correction algorithms are developed to improve the corrected photoacoustic imaging results, thereby enhancing the application value of photoacoustic human imaging in clinical diagnosis. The proposed motion-correction algorithms have been developed from manual, semiautomatic, to automatic real-time correction method, such as demons-based tracking and multi-scale vascular feature matching method [60], synthetic-surface-based algorithm [61], deep-learning-based [62], intensity phase tracking [63], and ultrasound-guided motion correction [59].

### 1.3 Clinical photoacoustic imaging platform and human application

**Fig. 2:**
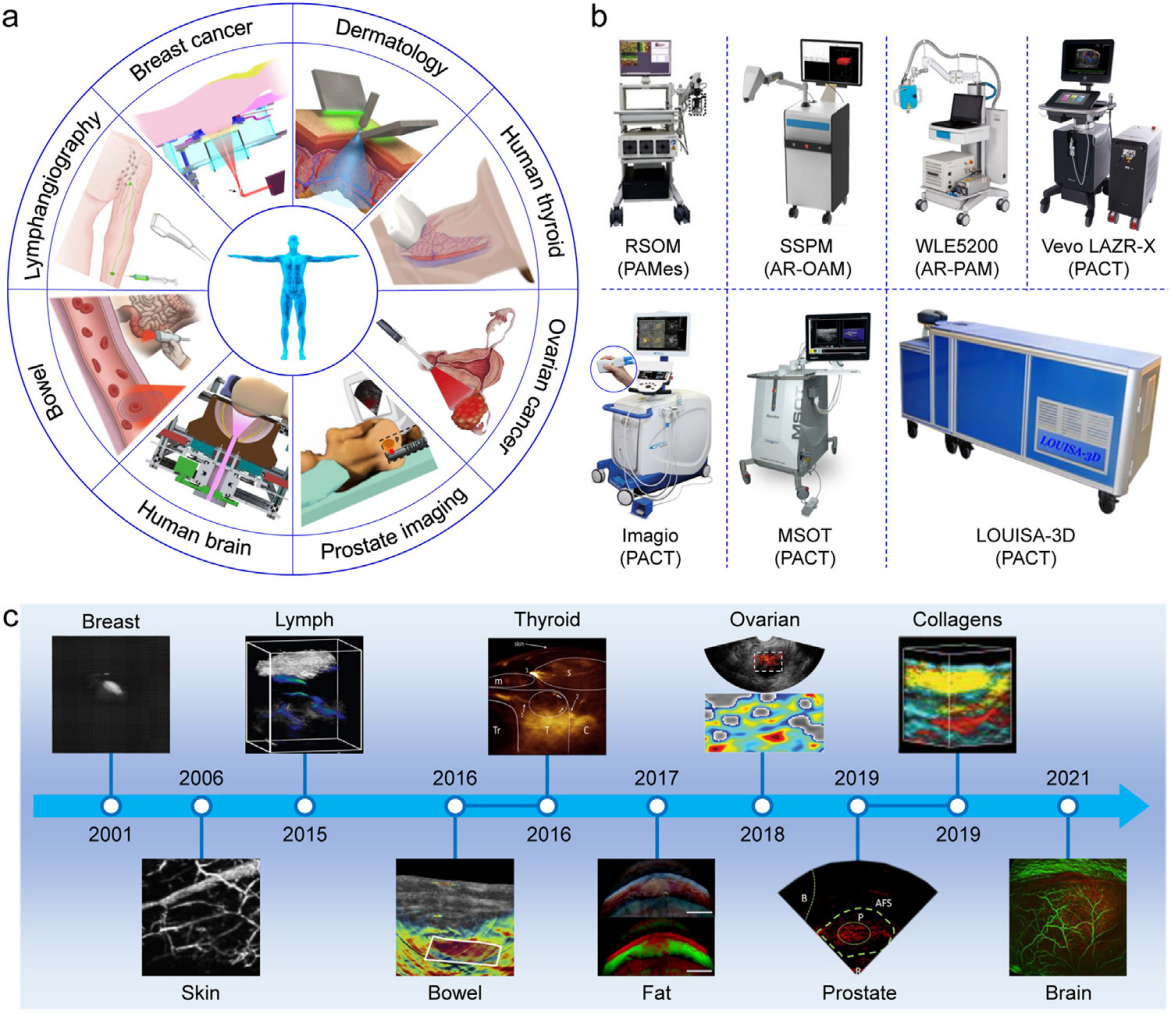
**Application of photoacoustic human imaging*****in vivo*****and representative commercial photoacoustic imaging platforms.** (a) Overview of photoacoustic human imaging *in vivo* [51,72,[74], [75], [76],^123^,^139^,^150^]. (b) Commercial photoacoustic imaging platforms. (c) The breakthrough researches and key applications of photoacoustic human imaging *in vivo* during the development of photoacoustics [64,71,73,74,76,92,123,130,145,147].

With the development of PAI technology, many medical equipment companies have begun to instrumentalize PAI systems, and have carried out photoacoustic human imaging based on the current situation of clinical imageology (Fig. 2a), aiming to enhance the level of clinical diagnosis. Representative companies include TomoWave Laboratories, iThera Medical GmbH, Seno Medical Instruments, FUJIFILM VisualSonics, CalPACT/Union Photoacoustic Technologies, Canon and Seno Medical Instruments, Mindray Bio-Medical Electronics, Guangdong Photoacoustic Medical Technology, etc. Current commercial PAM and PAMes devices (The first three platforms in Fig. 2b) are mainly used in clinical dermatology. In 2006, *in vivo* imaging of subcutaneous vasculature of the human palm was first performed [64], and then PAM and PAMes gradually played an important role in the diagnosis and treatment of skin diseases [51,[65], [66], [67]]. For example, iThera Medical GmbH has developed raster-scan optoacoustic mesoscopy (RSOM), which uses a customized fiber bundle to illuminate the human skin and receives PA signals through ultra-bandwidth ultrasound transducer. The RSOM system has successfully carried out pilot clinical study for psoriasis [51], atopic dermatitis [66] and skin sensitivity [68]. After achieving clinical translational application in the field of dermatology, PAI has gradually been used for the diagnosis of deeper organs. The imaging depth and field of view of PAM and PAMes do not allow them to cross the skin barrier to visualize subcutaneous organs [69]. However, PACT system provides superior field of view, deeper penetration, and faster imaging speed, which is suitable for the diagnosis of percutaneous organs (The PACT platforms in Fig. 2b) [13]. PACT usually combines fiber-bundle-based illumination with commercial clinical ultrasound probes to obtain the anatomic morphology and oxygen saturation of deep tissues. PACT technology has successively carried out research work in lymphatics [70], thyroid [71], breast [72], ovarian [73], prostate [74], intestinal imaging [75] as well as studies of brain function imaging *in vivo* [76] (Fig. 2c and Table 1). Currently, the most advanced system is massively parallel functional photoacoustic computed tomography for the human brain, which will provide a new perspective for brain science research. For commercial PACT platforms, multispectral optoacoustic tomography (MSOT) developed by iThera Medical GmbH can spectrally unmix different chromophores (for example, hemoglobin, lipids, melanin, collagens) [77]. Seno Medical's Imagio breast imaging system enables real-time evaluation to distinguish malignant from benign breast tissue. Notably, the Seno Medical received FDA premarket approval for its breast cancer diagnostic imaging device [78]. In China, Mindray Bio-Medical Electronics took the lead in completing the clinical translation of photoacoustic and ultrasonic dual-modality imaging technology for superficial organs [79]. There is no doubt that the emerging PAI is shining a new light on clinical cancer screening, diagnosis, and treatment [80,81].

**Table 1:** **Summary of*****in vivo*****photoacoustic human imaging introduced in this review.**

Human imaging in vivo	Application field	Current clinical techniques	Current clinical deficiencies	Biomarkers of PAI	Advantages of PAI
Breast	Breast tumor	X-ray mammography Ultrasonography MRI	Ionizing radiation Low contrast Expensive	Hb HbO_2_	High resolution Oxygen saturation Elastography
Skin	Skin disease Skin grafting Cosmetics	Dermoscopy RCM Ultrasound	Low penetration Low contrast	Hb HbO_2_ Melanin	High resolution Full-thickness skin Oxygen saturation Label-free
Lymph	Sentinel lymph nodes Lymph dissection Lymphangiography	Biopsy lymphography Lymphoscintigraphy	Repeat biopsy Radiation Complication	ICG	High resolution Vascular/lymphatic -colocalization
Bowel	Crohn's disease Rectal cancer	Endoscopy Ultrasonography CTE MRE	Low penetration Radiation Low contrast	Hb HbO_2_	Non-invasive Oxygen saturation Endorectal PAUS High diagnostic rate
Thyroid	Cancer Thyroid nodules	Fine needle aspiration Ultrasonography	Low contrast	Hb HbO_2_	High contrast Oxygen saturation
Fat	Fatty tumors	F-FDG-PET/CT MRI	Radiation Expensive	Lipid	Non-invasive Rapid imaging
Ovarian	Cancer	Transvaginal ultrasound	Low contrast	Hb HbO_2_	High contrast Oxygen saturation
Prostate	Cancer	Transrectal ultrasound	Low contrast	Hb HbO_2_ ICG	High contrast Simultaneous TRPA/US Specific imaging
Collagen	Bone health DMD	MRI	Expensive	Collagen	High contrast Non-invasive Rapid imaging
Brain	Brain function	fNIRS fMRI	Low-spatial-resolution Expensive	Hb HbO_2_	High resolution Functional imaging Rapid imaging

DMD, duchenne muscular dystrophy; RCM, reflectance confocal microscopy; CTE, computed tomography enterography; F-FDG-PET/CT, fluorodeoxyglucose positron emission tomography/computed tomography; fNIRS, functional near-infrared spectroscopy; fMRI, functional magnetic resonance imaging; TRPA/US, transrectal photoacoustic and ultrasound.

## 2 Advances in photoacoustic human imaging *in vivo*

### 2.1 Breast cancer diagnosis

PAI can non-invasively visualize the whole breast tissue with high spatial and temporal resolution, which has aroused great interest among researchers and clinicians worldwide. Breast cancer displaced lung cancer to become the most leading diagnosed cancer worldwide in 2020, and is also the leading cause of female cancer-related deaths [82,83]. Imaging plays an important role in the detection, diagnosis, staging, and response evaluation of breast cancer. At present, mammography has long been the mainstay of cancer screening and is the screening technology verified to reduce mortality, but X-ray examination is very painful, has adverse effects such as ionizing radiation, and the accuracy of diagnosis is reduced when the breast density is high [84]. Ultrasound imaging has speckle artifacts and low specificity in breast imaging [85]. Magnetic resonance imaging (MRI) brings a huge economic burden and requires the use of intravenous contrast agents, which is not suitable for mass screening or repeated examination [86]. In general, the existing clinical imaging technologies have significant advantages and limitations in the application of breast cancer. PACT is a hybrid optical imaging based on PA technology, which can reveal the distribution and oxygenation state of hemoglobin with higher spatial resolution, and obtain whole-breast structural and functional information through three-dimensional scanning [87], [88], [89].

**Fig. 3:**
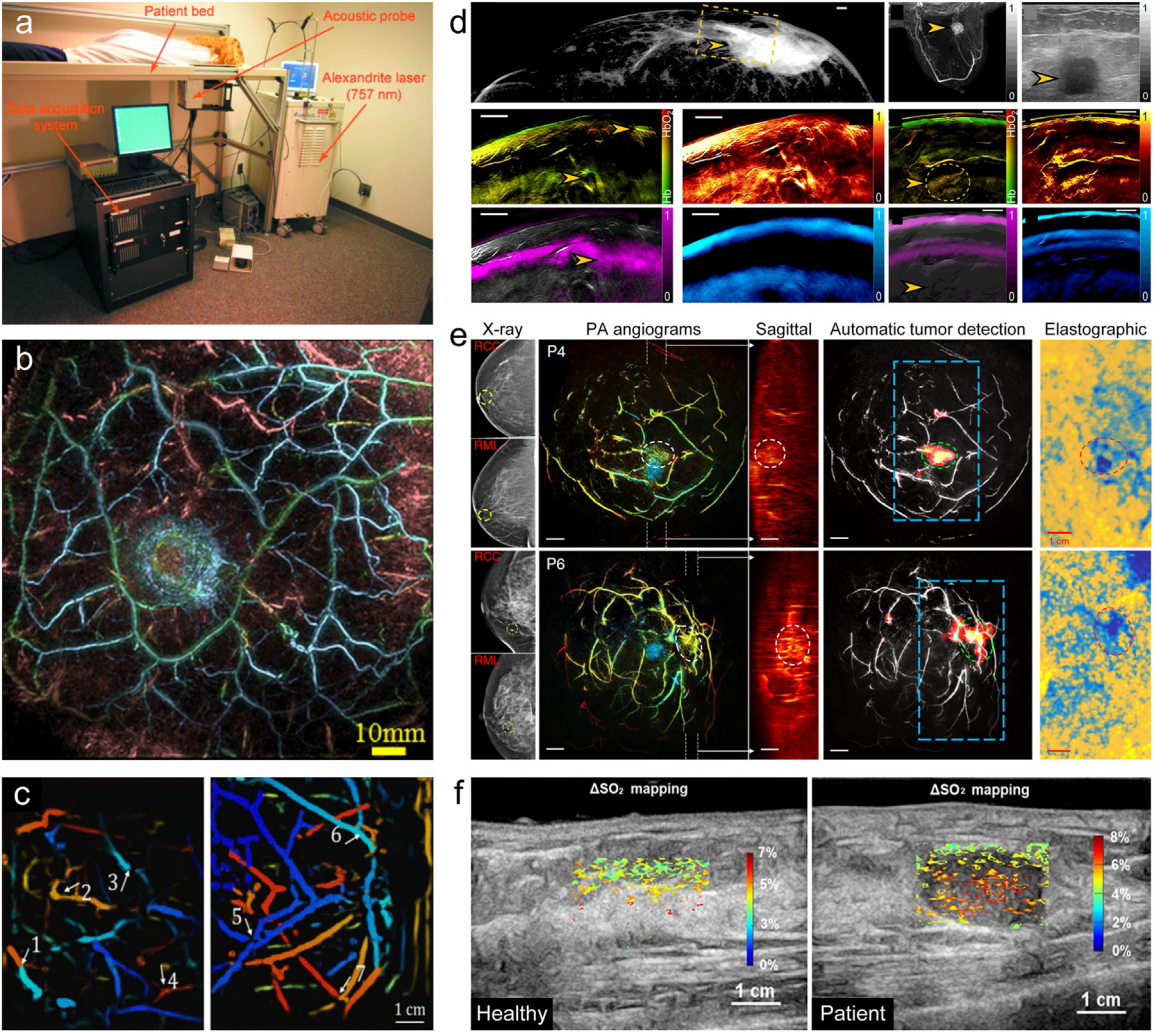
**Photoacoustic imaging of human breast.** (a) Clinical prototype LOIS-64 of photoacoustic breast imaging [93]. (b) Detailed morphological vascular characteristics on the healthy breast acquired by the PAI system with a hemispherical detector array [88]. (c) Imaging of a pair of breasts by a new portable photoacoustic breast imaging system [94]. (d) Multispectral photoacoustic tomography imaging of the largest and the deepest localized tumor [95]. (e) A single-breath-hold photoacoustic computed tomography imaging of cancerous breasts [72]. (f) Photoacoustic local SO_2_ distribution of healthy and patient volunteers [96].

After photoacoustic mammography was proposed, the theoretical research and the imaging system were quickly explored and developed [90,91]. In 2001, a clinical prototype of a laser photoacoustic imaging system was used for the first time in the detection and localization of breast cancer patients *in vivo* [92]. Clinical studies in breast cancer patients scheduled for surgical mastectomy were performed and compared with the conventional clinical methods. As an upgrade, Ermilov et al. developed the clinical prototype LOIS-64 (Fig. 3a) [93]. During the preliminary clinical studies on 27 patients, the LOIS-64 was able to visualize 18 out of 20 malignant lesions suspected from mammography and ultrasound images and confirmed by the biopsy after the PACT imaging. Further, Toi et al. developed a PAI system with a hemispherical-shaped detector array (HDA) [88]. As shown in Fig. 3b, the PAI system with HDA revealed finer vasculature and more detailed morphological vascular characteristics compared with MRI (Fig. 3b). What's more, vascular morphological abnormalities, including tumor-related centripetal vessels and disruption or narrowing of vessels, were diagnosed by PAI in breast cancer tissues as a result of the clinical study of 22 malignant cases. Nyayapathi et al. present a portable dual scan PACT system that provides visualization of angiographic features in a human breast with mammogram-like images (Fig. 3c) [94]. Diot et al. imaged 10 female patients aged 48–81 years with malignant nonspecific breast cancer or invasive lobular carcinoma by multispectral photoacoustic tomography [95]. Fig. 3d shows two cases from imaging of the largest tumor and the deepest localized tumor. Compared with conventional clinical imaging modalities, multispectral photoacoustic tomography resolves physiological cancer features with high resolution and deep penetration. Lin et al. developed a single-breath-hold photoacoustic computed tomography (SBH-PACT) system to reveal detailed angiographic structures in human breasts [72]. SBH-PACT features a deep penetration depth (4 cm *in vivo*) with high spatial and temporal resolutions (255 μm in plane resolution and a 10 Hz 2D frame rate). Higher vascular densities associated with tumors can be revealed at high spatial resolution, showing early promise for high sensitivity in radiographically dense breasts. In addition to angiography, the high imaging speed of SBH-PACT enables dynamic studies, such as photoacoustic elastography, which diagnoses tumors by showing less compliance. Imaging results of Patient 4 (P4) and Patient 6 (P6) were showed in Fig. 3e. The lesions in dense breasts are almost undiagnosable on the X-ray mammograms at P6. In contrast, SBH-PACT can clearly reveal tumors that are not easily seen on mammograms. The author then demarcated tumors in each breast and computed the average vessel densities inside and outside the tumors. The average vessel density ratios between the tumors and the surrounding normal breast tissues were 3.4 ± 0.99. Further, as showed in Fig. 3f, Han et al. calculated optical fluence distribution of breast tissue to increase the accuracy of local oxygen saturation [96], which helps to enhance clinical implementations of PAI. Most encouragingly, the photoacoustic breast imaging system of Seno Medical Instruments has been approved by the FDA for medical device marketing application [78]. PAI is expected to play an important role in early clinical diagnosis and screening of breast cancer.

### 2.2 Dermatological imaging

**Fig. 4:**
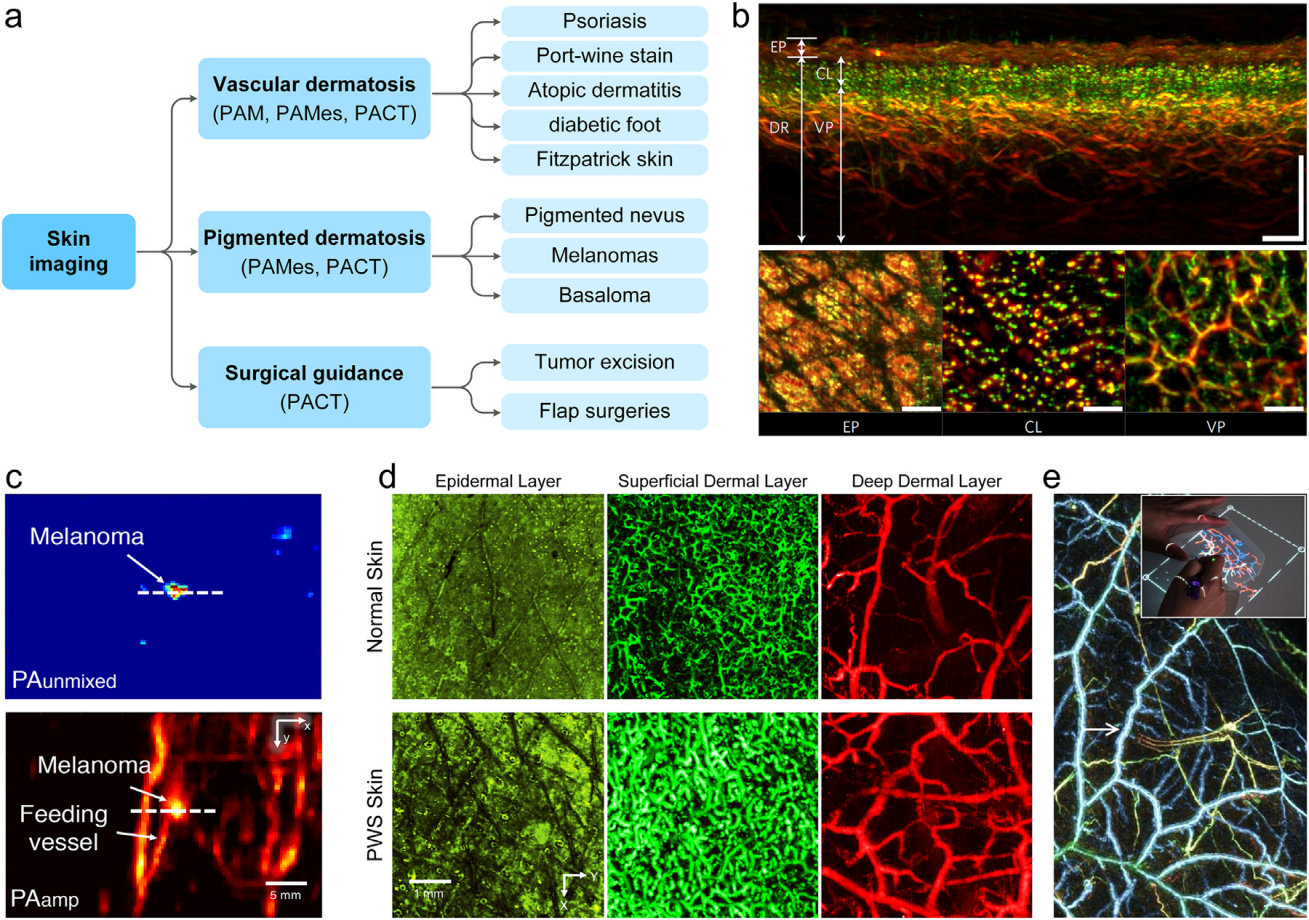
**Photoacoustic dermatologic imaging.** (a) Translational application of photoacoustic imaging in dermatology. (b) The fine structure of human skin is revealed by photoacoustic imaging [51]. (c) Photoacoustic imaging of human melanomas *in vivo*[52]. (d) Photoacoustic imaging of port-wine stain skin [65]. (e) Label-free photoacoustic imaging of human palmar vessels and anterolateral thigh flap [99,112].

The skin is the largest and most superficial organ of the human body. Human skin has complex multilayered structures with an average thickness of about 4 mm. Invasive biopsy-based pathological examination is still the gold standard for the diagnosis of skin diseases, but its shortcomings are also apparent. Non-invasive imaging technology plays a vital role in the diagnosis and treatment of skin diseases. However, the pure optical imaging technique is limited by the shallow imaging depth and can only visualize superficial skin tissues. Ultrasonic imaging technique can detect deep tissues, but it lacks detailed information on microscopic pathological structures. PAI can non-invasively obtain high-resolution anatomical structures by endogenous absorbers [97], [98], [99], such as melanin, hemoglobin, lipids, etc. In 2006, an *in vivo* volumetric image of the subcutaneous microvasculature of human palm was acquired using functional PAM [64]. The imaging system for human skin has gradually developed into photoacoustic dermoscopy (PAD) and has gained wide applications in dermatology (Fig. 4a) [98,[100], [101], [102]]. The emerging PAD can achieve full-thickness imaging of human skin [65,103] (Fig. 4b), revealing melanin in the epidermis, capillary loops in the epidermal-dermal junction layer, vascular network in the dermis, and big blood vessels in subcutaneous tissue. Berezhnoi et al. considered ultrawideband raster scan optoacoustic mesoscopy with an extended wavelength range from visible to short-wave infrared, and observed previously unseen high-resolution images of lipids colocalized with water, melanin, and hemoglobin distribution in human skin [104]. In pilot clinical study, PAD is gradually used in the detection and application of melanoma [52,105], psoriasis [51], port wine stains [97,106], diabetic feet [107,108], and cosmetic treatments [109]. Park et al. evaluated the use of 3D wide-field multispectral PAI to non-invasively measure depth and outline the boundary of melanomas for optimal surgical margin selection (Fig. 4c) [52]. The results provide well-measured depth and sizes of various types of melanomas, it also visualizes the metastatic type of melanoma. Obtaining accurate depth and boundary of melanoma before surgery would play a useful role in the complete excision of melanoma during surgery. Wang et al. developed a bifocal 532/1064 nm alternately illuminated PAM to capture deep vascular morphology in human skin [65]. The multilayered morphology in the port-wine stains skin can be used to accurately assess the lesion type rather than colorimetric assessment (Fig. 4d), in which case it enables clinicians to determine optimum treatment parameters for individual patients. For human skin imaging, some involuntary movements of the patient (such as breathing, arterial pulsation) will affect the accuracy of the acquired images. To be deployed in the clinic, PAD must be a robust technique. Aguirre et al. developed motion correction algorithms to help PAD fulfill its clinical promise [110,111]. For wider application in dermatology, Tsuge et al. demonstrated the utility of PACT for visualizing anterolateral thigh perforators in a clinical study of 10 thighs in 5 healthy adults (Fig. 4e) [112]. Evaluation of the correlation between PACT and ultrasound findings showed that PAT had comparable diagnostic potential but was superior in visualizing subcutaneous microvessels. Interestingly, they further transferred a PACT image to a body-attachable transparent sheet for real-time intraoperative navigation. There is no doubt that emerging PAI is opening new diagnostic prospects in the field of dermatology.

### 2.3 Lymphangiography

**Fig. 5:**
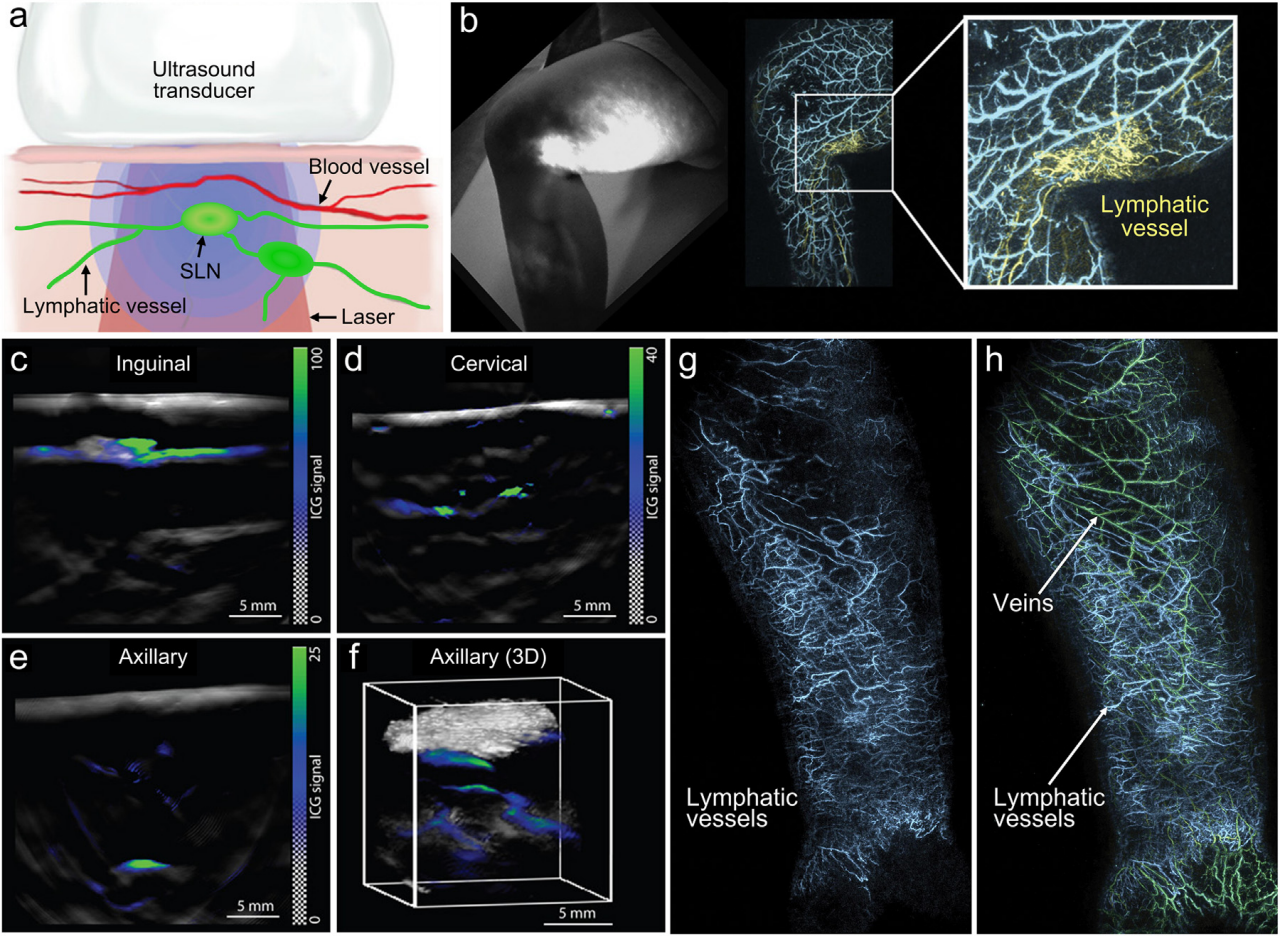
***In vivo*****photoacoustic lymphangiography.** (a) Schematic diagram of photoacoustic lymphangiography based on ICG [123]. (b) Photoacoustic lymphangiography evaluates the postoperative patency of lymphaticovenular anastomosis [124]. (c-e) Representative preoperative images of inguinal, cervical, and axillary SLNs from the 2D detector, with ICG signal overlaid on a single-wavelength background image at 800 nm. Images are from three different patients [123]. (f) Rendered view of an SLN imaged using the 3D MSOT detector, showing a network of lymph channels supplying the axillary lymph node of a different patient at ∼10 mm [123]. (g) dilated dermal lymphatics (blue) and, (h), superficial veins (green) [70].

The lymphatic system is an important defense function system in the human body [113,114]. The visualization of the lymphatic system is essential for the study of immune function. For example, sentinel lymph nodes (SLNs) act as barriers to the spread of tumor cells from the lymphatics, and SLNs biopsy has become a standard method for staging the disease in breast cancer patients [115]. Currently, the use of blue dye or radioactive imaging agents for SLNs biopsy, or ultrasound-guided fine needle biopsy cannot meet the clinical needs, and these techniques have ionizing radiation, invasive, and easy to cause complications. Moreover, the accuracy and specificity of intraoperative detection of SLNs are low. Therefore, there is an urgent need to develop an accurate, non-ionizing, and non-invasive method for detecting SLNs. As an emerging imaging technique, PAI has the advantages of high contrast imaging of endogenous and exogenous optical absorption and deep ultrasound imaging [116], [117], [118]. Due to the particularity of lymphatic vessels, endogenous absorbers such as hemoglobin cannot be used for PAI. Indocyanine green (ICG) is a Food and Drug Administration-approved contrast agent for fluorescence imaging and PAI (Schematic in Fig. 5a). Galanzha et al. performed detection, imaging, and flow cytometry of normal and cancer cells in lymph vessels and SLNs, including photoacoustic mapping of individual cancer cells in SLNs without (melanoma) and with (breast cancer) nanoparticles contrast agents [119], [120], [121]. Further, Kim et al. reported the feasibility of PAI with indocyanine green (ICG) for identifying SLNs and lymphatic vessels [122]. Stoffels et al. combined ICG and MSOT techniques to non-invasively detect the metastatic status of SLNs in melanoma [123]. MSOT technique significantly improved the tumor metastasis detection rate in excised SLN compared with the conventional clinical techniques. As shown in Figs. 5c-f, MSOT can demarcate the location of lymph nodes and the metastasis of lymph nodes with high resolution, and the deepest penetration depth can reach 5 cm. Kajita et al. used photoacoustic lymphangiography to evaluate the postoperative patency of lymphaticovenular anastomosis (Fig. 5b) [124]. The team also conducted a photoacoustic imaging of a patient clinically diagnosed with lymphedema with a history of malignant lymphoma with axillary lymph node dissection [70,[125], [126], [127]]. Photoacoustic lymphangiography was performed after subcutaneous injection of 0.5 mL ICG. For the first time, high-resolution 3D co-localized images of lymphatic and blood vessels are presented (Figs. 5g,h), showing a complex network of dilated lymphatic vessels and their relationship to the patient's right superficial veins of the forearm. Therefore, photoacoustic lymphangiography combined with ICG can more accurately visualize the lymphatic system with high resolution, helping to understand pathophysiology and evaluate cancer-related lymphatic diseases.

### 2.4 Intestinal imaging

**Fig. 6:**
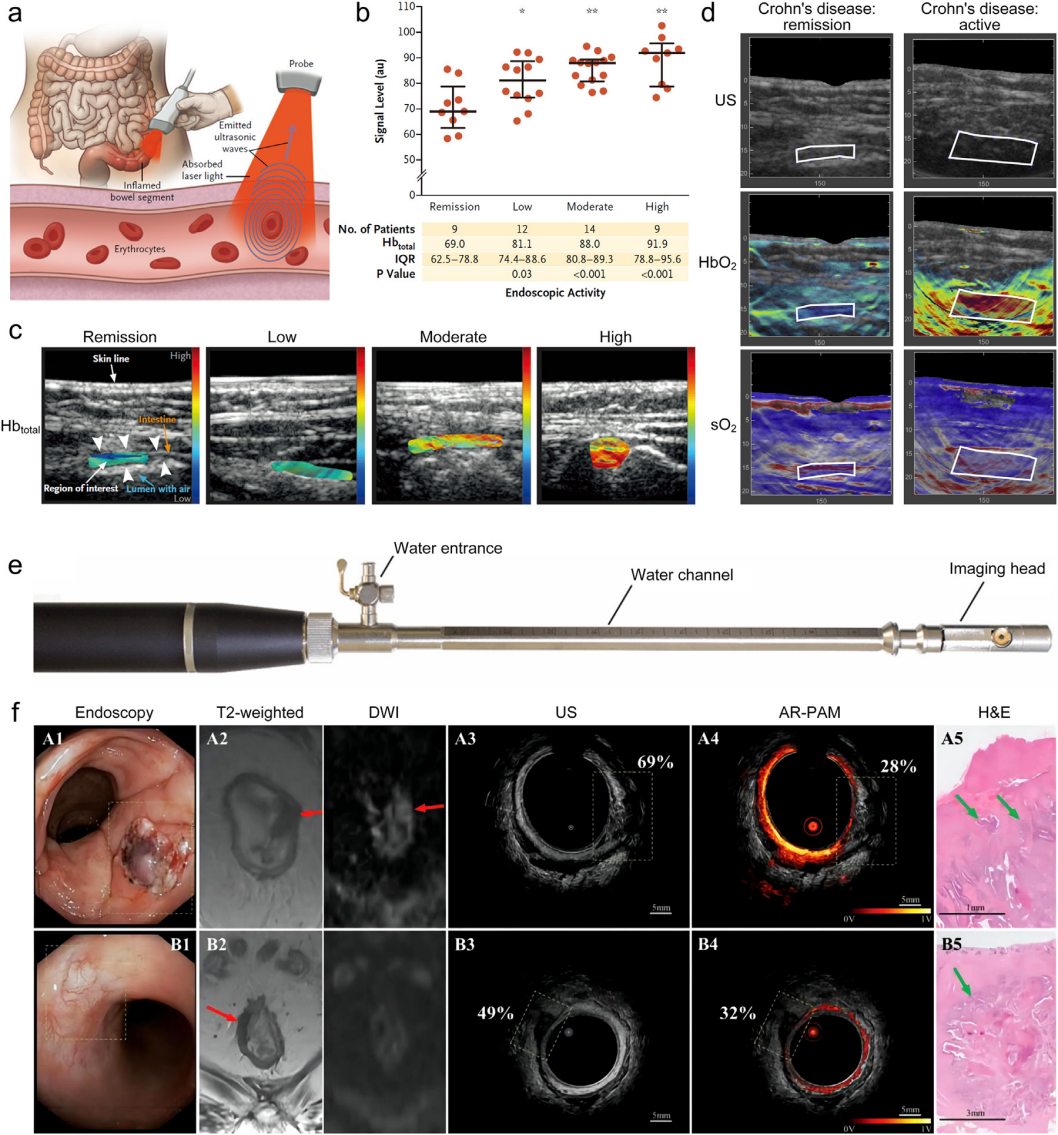
**Multispectral photoacoustic tomography and photoacoustic endoscopy for assessment of crohn's disease activity.** (a) Principle of non-invasive multispectral photoacoustic tomography for the evaluation of activity in Crohn's disease [75]. (b) Multispectral photoacoustic tomography evaluates the total hemoglobin [75]. (c) Multispectral photoacoustic tomography imaging of total hemoglobin [75]. (d) Non-invasive multispectral photoacoustic tomography for the evaluation of activity in Crohn's disease [130]. (e) Photograph of the endorectal coregistered photoacoustic microscopy/US imaging probe [132]. (f) Assess rectal cancer treatment response using the endorectal coregistered photoacoustic microscopy/US imaging paired with deep learning [132].

Inflammatory bowel diseases, such as Crohn's disease (CD) and ulcerative colitis, are caused by chronic recurrent inflammatory processes in the intestinal wall [128]. Acute exacerbations of inflammatory bowel disease require frequent adjustments or changes in treatment to reduce and prevent serious complications disease. Clinically, endoscopy and tissue biopsy evaluation are the gold standard methods for detecting bowel inflammation [129]. However, optical endoscopy can only visualize superficial tissue, resulting in a low diagnostic rate, and biopsy carries the risk of complications. At present, two branches of PAI technology, multispectral photoacoustic tomography (MSOT) and photoacoustic endoscopic (PAE) imaging, have been translated in gastrointestinal imaging. As a non-invasive deep imaging technique, MSOT can achieve transabdominal intestinal imaging, which can be used for non-invasive quantification of hemoglobin dependent blood perfusion and oxidation, as a surrogate indicator for evaluating enteritis. MSOT imaging can provide higher imaging contrast than conventional ultrasonography. Knieling et al. used commercial MSOT imaging system (iThera Medical) to perform transabdominal evaluation of intestinal inflammation with Crohn's disease (Figs. 6a-d) [75,130]. Six different wavelengths (700, 730, 760, 800, 850, and 900 nm) were used for MSOT data acquisition, and the total hemoglobin (Hb), oxygenated Hb, deoxygenated Hb, and oxygen saturation, are calculated from these measurements. Fig. 6b shows the total Hb signal levels detected by MSOT in the intestinal wall of 44 patients with Crohn's disease with different degrees of endoscopic inflammation. Further, imaging parameters of MSOT were confirmed by the simplified endoscopic score for Crohn's Disease (SES-CD). The results demonstrate that MSOT-based imaging of these parameters of the intestinal wall of patients with inflammatory bowel disease may be a highly sensitive and reliable method for non-invasive assessment of disease activity. As another branch of application, PAE imaging can be easily married with endoscopic ultrasonography, which can simultaneously obtain high-resolution absorption and structural information of the intestinal wall [131]. Leng et al. developed an endorectal coregistered photoacoustic microscopy/US (PAM/US) imaging system (Fig. 6e), paired with a convolutional neural network model, to enhance high diagnostic performance in assessing the rectal cancer treatment response [132]. The used PAM/US endorectal probe consisted of an endorectal imaging probe, a 1064-nm laser, and one US ring transducer. The PAM convolution neural network and US convolution neural network models were trained and validated to distinguish normal from malignant colorectal tissue using *in vivo* patient data (Fig. 6f). Qualitatively, PAUS images of normal colorectal tissue show layered rectum wall structure and uniformly distributed submucosal vasculature, whereas malignant tissue showed distortion of this orderly layered pattern and obliteration of submucosal vasculature within lesions. The PAM convolution neural network model captures this unique vasculature signature for automated data processing from a large volume of PAM/US data and differentiates among residual tumors, normal rectum tissue, and the tissue of treatment responders. Current clinical advances suggest that PAI can be used as a novel approach for rapid, non-invasive assessment of intestinal diseases.

### 2.5 Thyroid imaging

**Fig. 7:**
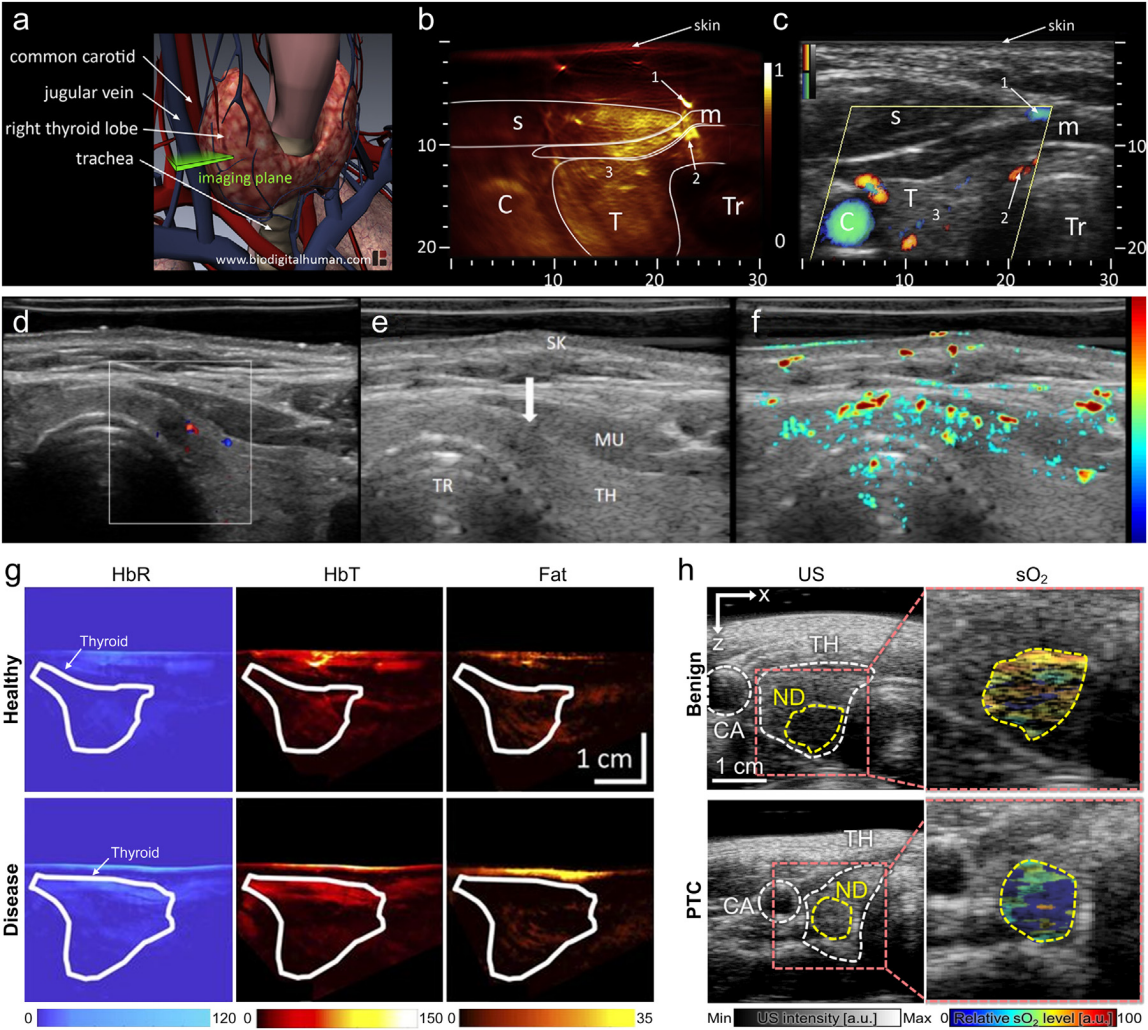
**Photoacoustic and ultrasound imaging of the thyroid.** (a) Anatomy of thyroid gland including cardio-vascular and respiratory system; the cross-sectional 2D imaging plane is highlighted in green. (b-c) Photoacoustic and ultrasound cross sections of the left thyroid lobe of the first volunteer [71]. C: Carotid, T: Thyroid, Tr: Trachea, s: sternocleidomastoid muscle, m: infrahyoid muscle; axes in mm. Color Doppler flow imaging ultrasound (d), gray scale US B-scan (e) and PA/US (f) imaging results for a left lobe papillary thyroid cancer (PTC, Follicular variation) with the largest diameter of 3 mm [138]. (g) Example pseudo color-coded MSOT images of HbR, HbT, and fat of Graves’ disease and healthy thyroid tissue [139]. Images show higher HbR and HbT and lower fat content in Graves’ disease than in healthy tissue. ROIs transferred from corresponding ultrasound images are shown to localize investigated thyroid lobe. (h) The *in vivo* PAUS images and multispectral PA signals acquired from one benign patient and one PTC patient patients [140].

The incidence of thyroid cancer is on the rise, and this disease is projected to become the fourth leading type of cancer across the globe [133]. The rise in global incidence has been attributed to several factors, including increased over-diagnosis, individual risk factors and increased exposure to environmental radiation [134,135]. Ultrasound-guided fine-needle aspiration biopsy of thyroid is currently the gold standard for the diagnosis of thyroid cancer [136]. However, due to the limitation of the sampling site, the tumor cannot be fully observed, which leads to repeated biopsies and unnecessary surgery, and even causes inflammation or cancer. Current clinical imaging techniques, such as MRI, CT, PET/CT, etc., lack specificity and cannot accurately distinguish benign and malignant follicular nodules [137]. The thyroid is in the superficial 2∼3 cm depth range, so PAI has great potential in clinical diagnosis and treatment of the thyroid nodule. Non-invasive specific diagnostic analysis of PAI can reduce unnecessary biopsies, avoid wasted medical costs and improve the patient's quality of life. Dima et al. developed a compact handheld version of real-time MSOT platform using a detector adapted to the dimensions and overall geometry of the human neck, to demonstrate the feasibility of MSOT imaging for the human thyroid for the first time [71]. As shown in Figs. 7a-c, the feasibility of hand-held thyroid MSOT platform was evaluated on healthy human volunteers. The results were compared with ultrasound and Doppler ultrasound images obtained from the same volunteers. The imaging results demonstrate that the MSOT can accurately resolve optical absorption consistent with the anatomy and morphology of the thyroid. Yang et al. developed an initial clinical study of *in vivo* human thyroid by a PA/US handheld probe based on a high-end clinical ultrasound machine [138]. In Yang’ study, both healthy and cancerous thyroids were imaged non-invasively, and the PA results were compared with Doppler ultrasound. Figs. 7d-f show that PA thyroid imaging could reveal detailed blood vessels that were not sensitive for Doppler ultrasound. Roll et al. used MSOT platform to study thyroid diseases, including Graves' disease and thyroid nodules (Fig. 7g) [139]. Quantitative analysis of deoxyhemoglobin, total hemoglobin, and fat content further demonstrated the applicability and potential of MSOT in improving non-invasive diagnostics of thyroid disease. Kim et al. performed multiparametric analysis of multispectral PA data of thyroid nodules (Fig. 7h) [140]. They performed *in vivo* multispectral PA imaging on thyroid nodules from 52 patients, comprising 23 papillary thyroid cancer (PTC) and 29 benign cases. From the multispectral PA data, they calculated hemoglobin oxygen saturation level in the nodule area, then classified the PTC and benign nodules with multiparametric analysis. Statistical analyses showed that this multiparametric analysis of multispectral PA responses could more accurately classify PTC nodules.

### 2.6 Collagen and fat imaging

**Fig. 8:**
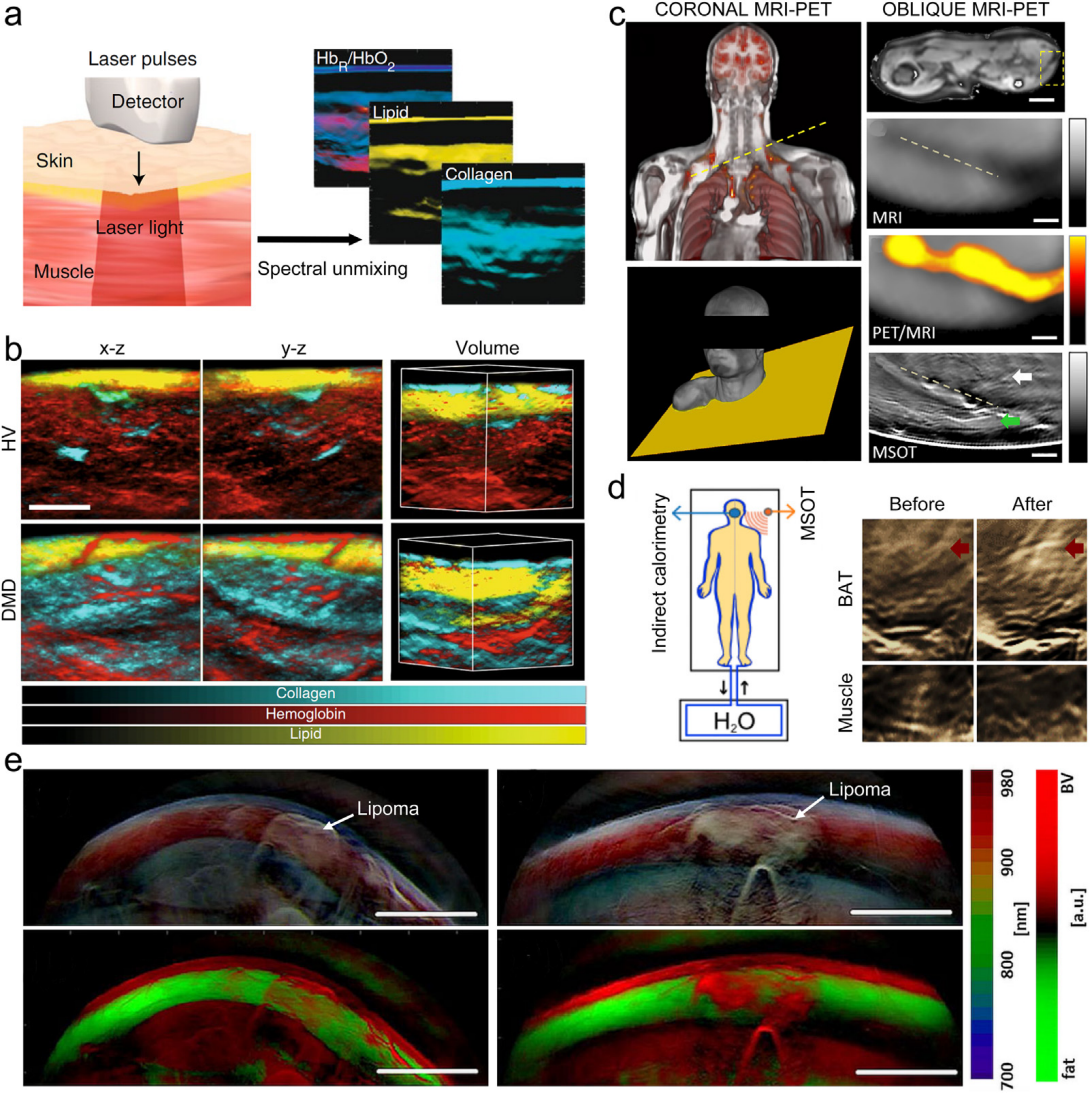
**Multispectral photoacoustic imaging of collagen and fat.** (a) Different chromophores can be separated by spectral unmixing based on the specific absorption and reflection properties of the emitted light [145]. (b) *In vivo* 3D multispectral photoacoustic imaging of healthy volunteers and patients with Duchenne muscular dystrophy [145]. (c) MRI-PET coronal co-registration of the upper torso of a volunteer; the MSOT imaging plane is indicated by the yellow dotted line. MSOT handheld image, showing BAT (white arrow) and muscle (green arrow) [146]. (d) Schematic of simultaneous indirect calorimetry and MSOT measurements of cold activation using 13 °C water before and after cold activation. MSOT was used to image BAT (red arrows) and muscle areas with white and green arrows, respectively [146]. (e) Imaging of fatty tumors: appearance of subcutaneous lipomas in photoacoustic images [147].

Collagen and fat are important endogenous components of the human body and are closely related to human health, and they can be used as biomarkers for monitoring disease progression and treatment response [141]. For example, collagen is an important biomarker of osteoporosis progression and Duchenne muscular dystrophy [142], and adipose tissue plays a key role in understanding metabolic disorders [143]. MRI and magnetic resonance spectroscopy have shown potential as non-invasive imaging to quantify collagen and fat. However, their applicability is limited due to the rather long acquisition time. MSOT imaging can rapidly and non-invasively realize spectral unmixing of endogenous chromophores (Fig. 8a) [77,144], and realize high-resolution 3D quantitative imaging of collagen and fat, which is expected to be used for disease monitoring and treatment evaluation. Regensburger et al. studied collagen using MSOT imaging, in which near-infrared illumination at 680–1100 nm was used for visualization and quantitative analysis of collagen content *in vivo* [145]. First, the feasibility of MSOT for non-invasive quantification of tissue fibrosis was demonstrated in a longitudinal study in a large animal model of Duchenne muscular dystrophy, and then MSOT imaging was applied in pediatric patients. The results demonstrate that collagen content in skeletal muscle obtained by MSOT imaging is highly correlated with functional status in patients with Duchenne muscular dystrophy, and that MSOT imaging provides more information on molecular features than MRI. Fig. 8b shows 3D MSOT images of two boys with high resolution and clear visualization of subcutaneous chromophores (collagen, hemoglobin, lipid). Significant differences were found in endogenous chromophores in the tissues of healthy volunteers and Duchenne muscular dystrophy patients. In another study, Reber et al. used video-rate handheld MSOT platform with the 700–970 nm spectral range to non-invasive visualize BAT activation [146], the neck and supraclavicular regions of volunteers were imaged (Figs. 8c,d), and the MSOT imaging results were validated using conventional imaging techniques. MSOT images provide a more detailed imaging of the tissue than PET or MRI. The MSOT images capture information corresponding to a triangular muscle structure, which is also visible in the oblique MR image, but at much lower resolution. Furthermore, differences in brown adipose tissue between healthy and diabetic tissue were observed in this study. The findings demonstrate that MSOT imaging can be used as a useful tool to study the fundamentals of adipose tissue metabolism, and it can help further study the role of adipose tissue in the management of obesity and diabetes. Further, the hand-held MSOT platform was used to image superficial fatty tumors (Fig. 8e) [147], and the MSOT images were compared with diagnostic ultrasound. Fatty tumors were clearly visualized by MSOT and exhibited a spectral signature which differed from normal fatty tissue or muscle tissue with high contrast. Based on preclinical validation, MSOT is expected to have promising applications in the diagnosis and evaluation of subcutaneous soft tissue masses.

### 2.7 Ovarian and prostate imaging

**Fig. 9:**
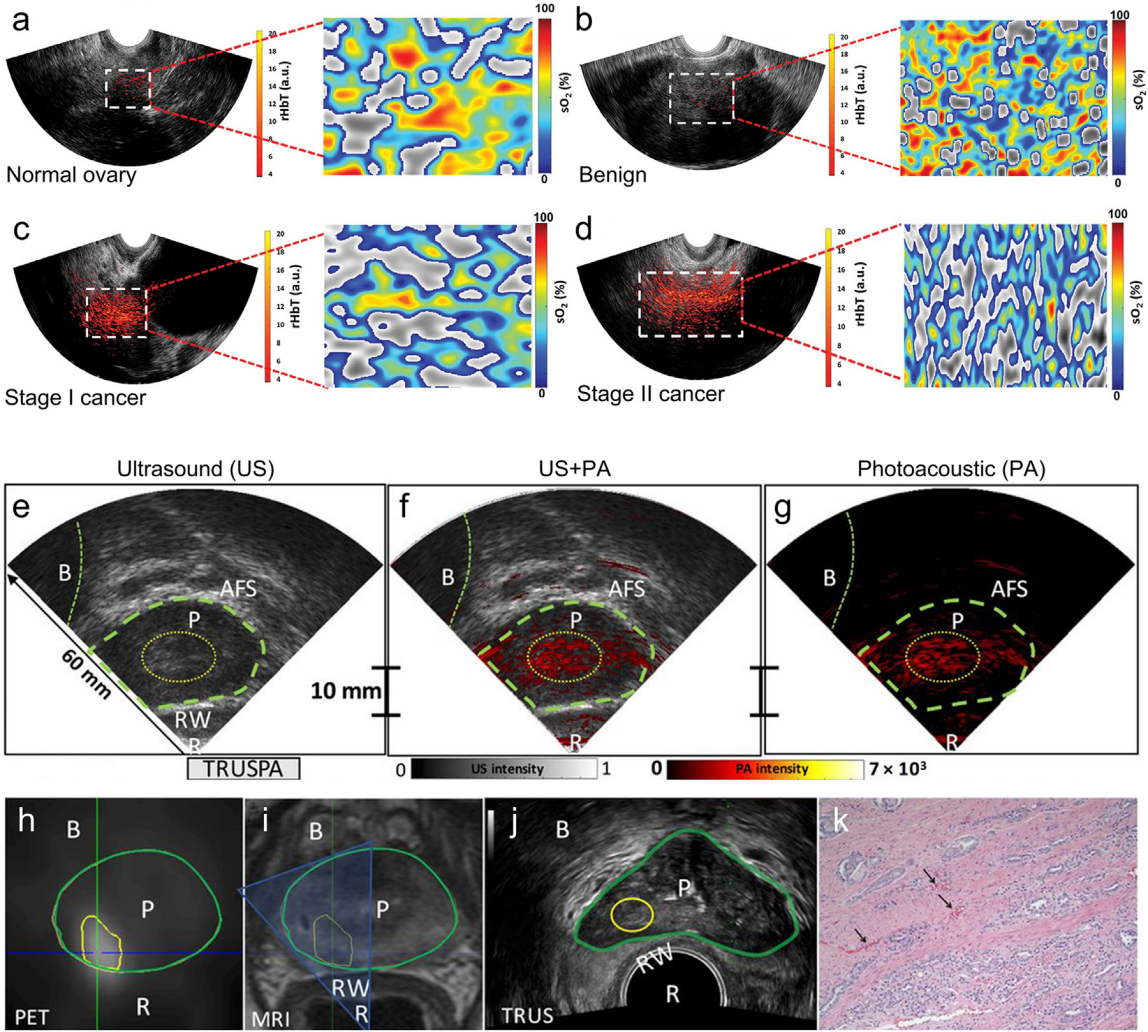
**Photoacoustic tomography imaging of ovarian cancer and prostate cancer.** (a-d) Transvaginal photoacoustic tomography and pulse-echo US imaging of normal ovary, benign fibrothecoma, stage I endometrioid adenocarcinomas, and stage II endometrioid adenocarcinoma [73]. (e-g) Transrectal ultrasound and photoacoustic imaging of the prostate in a patient with prostate cancer [74]. (h-k) PET, MR, transrectal ultrasound, and histopathology imaging [74].

At present, ovarian cancer is still the deadliest of all gynecological malignancies [148]. Due to the lack of effective imaging technology, only 20% to 25% of ovarian cancer can be diagnosed at an early stage. Although imaging methods such as CT, PET, and MRI have been used for surgical guidance, shortage of promising screening tools for early-stage detection remains one of the major challenges linked with the poor survival rate for patients with ovarian cancer. There is an urgent need to develop effective ovarian tumor imaging tools to obtain quantitative information such as oxygen saturation, hemoglobin tissue level, and blood flow. PACT can provide high-resolution structural and functional information ranging from several millimeters to several centimeters in depth. Nandy et al. explored the diagnostic value of transvaginal coregistered PACT and pulse-echo US imaging for ovarian cancer [73,149,150]. In this study, 26 ovarian masses from 16 participants were successfully imaged *in vivo*, including nine invasive epithelial ovarian cancers, three other tumors, and fourteen benign and normal ovaries. The relative total hemoglobin concentration (rHbT) and mean oxygen saturation (sO_2_) were used to diagnose and evaluate the ovaries. The results (Figs. 9a-d) showed that the rHbT was 1.9 times higher for invasive epithelial cancers than for the benign/normal ovaries (*P* = .01). The mean sO_2_ of invasive epithelial cancers, and of the borderline and stromal tumors, was 8.2% lower than that of benign/ normal ovaries (*P*=.003). Invasive epithelial ovarian cancers showed higher and extensive tumor vascularity and lower sO_2_ than benign and normal ovaries. This study demonstrates that PAI holds great potential to improve the diagnosis of ovarian cancer.

As a similar disease, prostate cancer is the most common non-cutaneous cancer in men, with more than 1 million new cancer cases worldwide and more than 300,000 deaths each year. Since the 1980s, transrectal ultrasound (TRUS) has been routinely used to visualize prostatic anatomy and guide needle biopsy, but ultrasound imaging has limited specificity and cannot provide anatomical, functional, and molecular information at the same time. Gambhir et al. developed an integrated transrectal ultrasound photoacoustic (TRUSPA) device that simultaneously visualized the anatomy of the prostate in human subjects using ultrasound images and observed functional and molecular optical comparisons using photoacoustic images [74]. The study achieved multispectral PA imaging of the contrast between hemoglobin and ICG within the range of 3–4 cm in the human prostate. Figs. 9e-k present *in vivo* TRUSPA imaging results from a patient with proven prostate cancer as evidenced on preoperative PET-MRI, using 68 Ga labeled PET tracer targeting of bombesin on the prostate cancer cells, and followed by PET-MRI contrast-targeted biopsy (with MRI-TRUS fusion) of the prostate using the conventional TRUS device. In agreement with the PET-MRI results, the TRUSPA device displayed a distinct PA contrast from the right peripheral base of the prostate, which was not present when scanned through other prostatic regions of this patient. The results confirmed that the ingenious combination of PAUS is expected to enrich and improve the diagnostic capabilities of current clinical ultrasound examinations.

### 2.8 Functional brain imaging

**Fig. 10:**
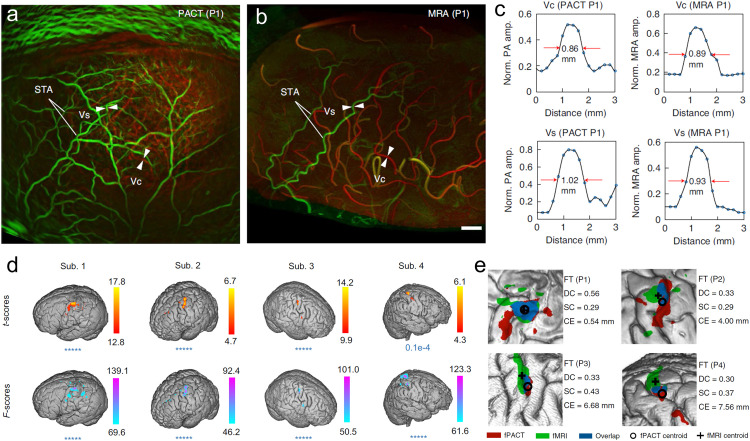
**Massively parallel functional photoacoustic computed tomography of the human brain**[76]**.** (a) Photoacoustic computed tomography angiography of human brain. (b) Magnetic resonance angiography of the same brain. (c) The diameters of the selected scalp vessel (Vs) and cortical vessel (Vc) of participant 1 were quantified as the full width at half maximum (red arrows). (d) Functional responses to sequential finger tapping were recorded in participants 1–4 by photoacoustic imaging. (e) The functional maps of sequential finger tapping.

Functional imaging of human brain is one of the important fields of clinical medical imaging technology applied in neuroscience [151,152]. Functional brain imaging plays an important role in the study of pathogenesis, prevention and treatment strategies of functional brain diseases, and provides important auxiliary information in clinical diagnosis and treatment of brain diseases [153]. The advent of MRI has provided tools to study *in vivo* human brain imaging, and further, blood oxygen level-dependent (BOLD) functional magnetic resonance imaging (fMRI) makes functional brain imaging *in vivo* a reality [154,155]. It studies cortical activity of the brain by using changes in the ratio of oxygenated hemoglobin to deoxygenated hemoglobin in blood. fMRI can achieve sub-millimeter/sub-second spatiotemporal resolution, but its cost is very high, and the equipment is bulky and unsuitable for claustrophobic patients. Moreover, fMRI is only suitable for group-level analysis, how to apply it to the individual level is still a challenging problem [156,157]. PAI can non-invasively visualize the vasculature of the human brain through the light absorption properties of endogenous hemoglobin [158], so it can study the brain neural activity specifically based on the neurovascular coupling. Compared with fMRI, PAI can map cerebral blood vessels with high sensitivity [159], [160], [161], [162], and further MSOT imaging can use spectral features to quantify the characteristics of blood oxygen saturation and cerebral blood volume [162]. PAI technology is expected to become a major player in the field of neuroimaging and neuroscience, filling a gap between existing techniques in terms of portability, spatial and temporal resolution. Na et al. designed an *in vivo* 3D functional human brain PACT system of 1024 (1 K) parallel ultrasonic transducer elements, termed 1K3D-fPACT [76]. Compared with conventional PACT systems, it has a larger field of view and faster imaging speed. The 1K3D-fPACT system has a spatial resolution of 350 μm and a temporal resolution of 2 s. For the first time, photoacoustic imaging of the human brain has been achieved. Fig. 10a and Fig. 10b show the results of PACT angiography and magnetic resonance angiography (MRA) of the human brain, respectively. MAR is mainly sensitive to arterial vessels, whereas PACT is sensitive to hemoglobin and visualizes both arteries and veins. Quantitively, the measured vasculature diameters exhibit a strong agreement (Fig. 10c). Vascular imaging validates potential of developed fPACT for diagnosing cerebrovascular disease. For functional brain imaging, Fig. 10d,e show that fPACT recorded functional responses to sequential finger tapping were recorded in participants. In this experiment, functional imaging at a depth of 1 cm from the cerebral cortex was achieved by being directly sensitive to hemoglobin. The presented results demonstrate that the emerging fPACT technology is likely to be a robust, powerful, and practical tool for human neuroimaging in the future.

### 2.9 Other human applications

In addition to the imaging applications of photoacoustic techniques in the above-mentioned human body parts or organs, photoacoustics has also yielded impressive results in other human cases. For example, rheumatoid arthritis can be diagnosed by dual-modality photoacoustic and ultrasound imaging technique. Multiple parameters such as relative concentration of total hemoglobin, oxygenation status, ratio of angiogenesis, joint size and area of synovia can be obtained to assess the development and treatment of rheumatoid arthritis [163], [164], [165]. As a new paradigm for understanding vascular health, photoacoustic imaging is used to monitor peripheral blood vessels in humans [166]. Photoacoustic imaging can also be used to detect periodontitis and monitor gingival health [167]. For another, as a new diagnostic method, photoacoustic spectroscopy can be used for cancer therapy, renal failure, diabetes mellitus, autism, schizophrenia, and more [168], [169], [170].

## 3 Directions for enhancing imaging

**Fig. 11:**
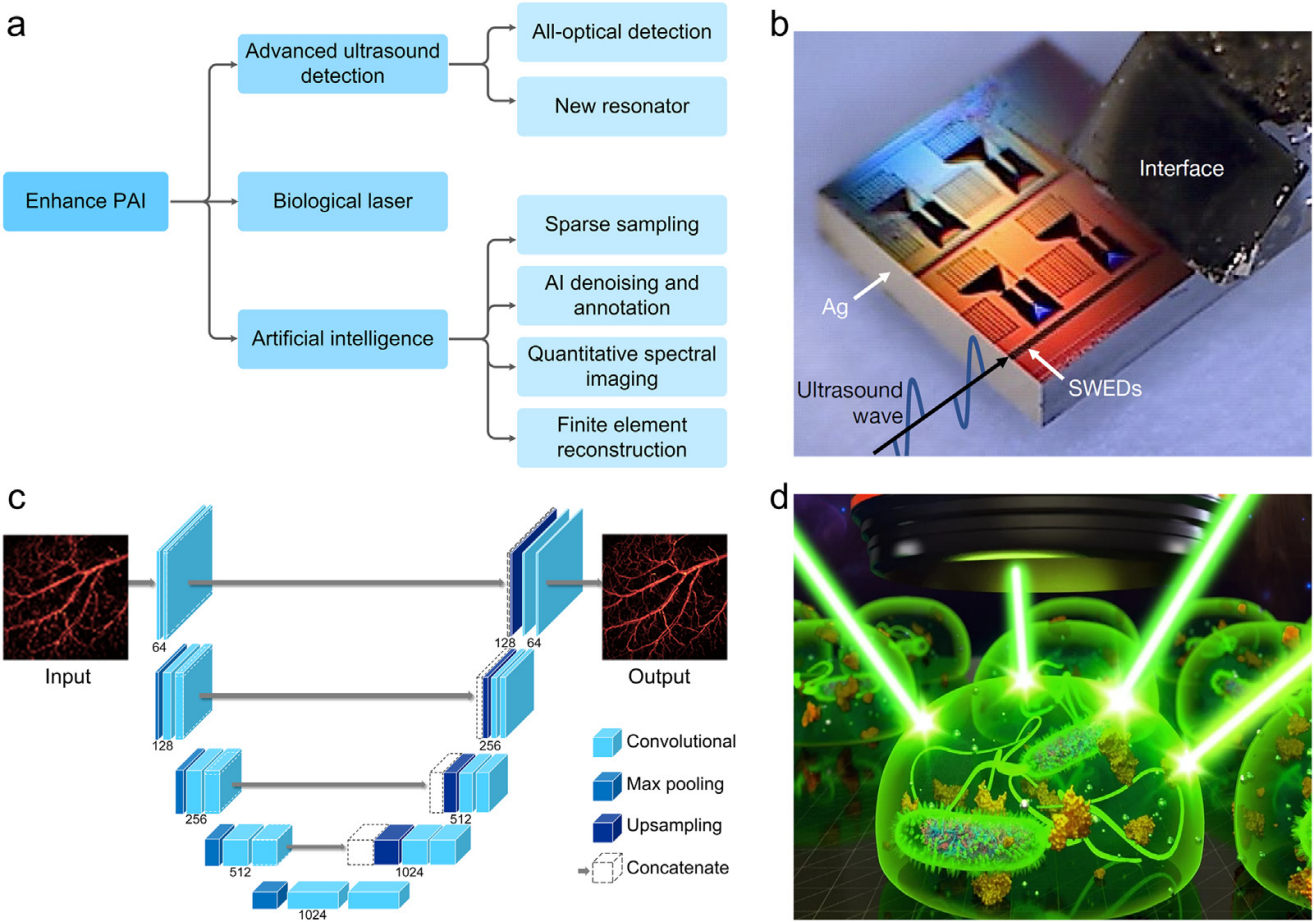
**Future advanced research directions for photoacoustic imaging.** (a) Directions are proposed to enhance photoacoustic human imaging. (b) Advanced ultrasound detection [171]. (c) Deep learning photoacoustic imaging with sparse data. (d) Biolaser array [177].

Photoacoustic human imaging is a promising biomedical imaging technique based on the absorption of endogenous or exogenous contrast agents, combining the rich contrast of optical imaging with the deep penetration of ultrasonic imaging. Endogenous chromophores contained in the human body have strong light absorption in specific optical windows, which can provide high photoacoustic contrast, such as nuclei (a peak at ∼266 nm), melanin (broadband absorption), hemoglobin (visible-light window), lipid (a peak at ∼930 nm), collagen (a peak at ∼1725 nm), etc. To further expand the application scenarios and enhance the performance of PAI, some exogenous contrast agents have been used in clinical (preclinical) applications. Generally, these contrast agents have absorption peaks in the long wavelength range for deeper imaging depth, such as indocyanine green (a peak at ∼808 nm), methylene blue (a peak at ∼664 nm), etc. In addition, some emerging carbon-based nanomaterials, small organic molecules, and semiconductor polymer nanoparticle contrast agents have been proposed and developed to enhance sensitivity, specificity, and efficiency of deep tissue imaging *in vivo*. Although preliminary human imaging experiments and preclinical imaging studies have shown the great clinical potential of PAI, the only devices currently approved for clinical use by the Food and Drug Administration are breast and skin imaging devices, indicating that PAI still faces many challenges in practical clinical applications. As a natural hybrid imaging technology, the development of PAI technology is particularly dependent on the development of multi-disciplines, such as materials science, optics, acoustics, and computer science. To achieve the full potential of photoacoustic clinical application, the further advances in multidisciplinary are required to better carry out clinical applications. In the future, the development of advanced ultrasound detection [35,171,172], and artificial intelligence technology [173], [174], [175], and biological laser [176], [177], [178], [179] is expected to release the application prospect of clinical photoacoustic human imaging and accelerate the translational applications of technology for PAI (Fig. 11a).

### 3.1 Advanced ultrasound detection

With the development of acoustics and material science, the detection technology of PA signal will accelerate the translational applications of PAI platform in clinic. For example, with the vigorous development of CMUT/PMUT technology and transparent transducers [28,[180], [181], [182]], PAI platform is expected to develop towards miniaturized, flexible and wearable devices, which is of great significance for long-term monitoring of chronic diseases such as diabetic foot. All-optical photoacoustic detection technology has the advantages of non-contact and high sensitivity, which can expand the application scenarios of PAI [35,183]. Notably, the developed sub-micron silicon chip ultrasonic detector with a sensing area of only 220 nanometers by 500 nanometers by scientists has highly sensitive and super-resolution imaging performance (Fig. 11b) [171], which has great potential to be implanted into brain tissue to detect acoustic signals, which is expected to be an important imaging field for brain science research.

### 3.2 Artificial intelligence

The development of artificial intelligence technology will help PAI technology to be better used in clinical human application. As shown in Fig. 11c, sparse sampling technology based on deep learning can improve imaging speed while ensuring resolution, and deep learning can play an important role in denoising, photoacoustic spectroscopy quantitative imaging, compensating and correcting motion artifacts, and enhancing the quality of photoacoustic image reconstruction based on finite receiving angle [62,[184], [185], [186]]. In addition, artificial intelligence technology can also be used to determine the boundary between tumors and normal tissues, providing accurate information for clinical diagnosis and treatment [187]. Artificial intelligence technology will play an important role in promoting photoacoustic human imaging in clinical diagnostics.

### 3.3 Biological laser

In 2011, the biological cell laser based on green fluorescent protein was successfully realized for the first time [188], which opens a new window for tissue imaging within biological systems. Biological laser is a frontier research direction at the intersection of biophysics and life sciences [189]. Although it is still in the early stage of development and the single pulse energy is small, it still has great potential to carry rich histological information for clinical imaging technology. Through the biocompatible cell laser technology that can be implanted in the human body, the acoustic information at the cellular level in the body can be obtained to enhance the non-invasive diagnosis of clinical diseases. Imaging-based optofluidic biolaser array is expected to be used for rapid and precise localization of lesions (Fig. 11d) [177]. *In vivo* biological laser excitation can realize *in vivo* study of deep human tissues with cell-scale resolution, which has great potential for studying cell activities, exploring carcinogenesis causes, and accurately determining cancer lesions [190]. The development of biological laser will undoubtedly promote PA diagnostic imaging technology to better serve the field of life science.

## 4 Conclusion

The purpose of this review is to show the breakthrough researches and key applications of photoacoustic human imaging *in vivo*, especially for clinical disease diagnosis, providing clinical translational orientations and for photoacoustic community and clinicians. We reviewed different configurations of the PAI platform, namely PAM, PAMes and PACT, and introduced the system structures, imaging parameters, translational progress and clinical prototype development in detail. Now, the potential of PAI in translational applications has been demonstrated by the first FDA approval of this technology for breast cancer screening. We focused on *in vivo* photoacoustic human imaging, which opens an entirely new window for the precise diagnosis and treatment of clinical diseases. For example, PAI is used for early screening and quantitative evaluation of cancer, precise guidance of skin grafting, detection of new disease markers, brain functional imaging research and so on. PAI has some significant advantages over conventional clinical imaging techniques. Firstly, PAI is a non-ionization, non-radiation, and non-destructive imaging technology, which fills the spatial resolution gap of pure optical and pure acoustic imaging technology, and makes up for the blind spot of existing clinical imaging technology. Secondly, label-free multispectral photoacoustic imaging enables accurate three-dimensional analysis of different tissue components and oxygen saturation measurement. In addition, PAI is a highly sensitive absorption imaging technology, which utilizes the endogenous chromophores in human biological tissues to achieve deep and high-contrast imaging. What's more, as an emerging hybrid imaging technique, PAI is naturally married with conventional clinical imaging techniques to integrate optical, acoustic and magnetic multimodal imaging technology, providing comprehensive morphological structures and multifunctional physiological conditions, and thus improving the diagnostic ability of clinicians. Overall, PAI is playing an emerging role in clinical diagnostics and is opening a new horizon for life science research.

## Declaration of competing interest

The authors declare that they have no conflicts of interest in this work.
